# Vitreoretinal interface features in epiretinal membrane associated with pseudoexfoliation syndrome

**DOI:** 10.1007/s00417-025-06854-1

**Published:** 2025-05-21

**Authors:** Denise Vogt, Viktoria Deiters, Yulia Zaytseva, Ricarda G. Schumann, Armin Wolf, Siegfried G. Priglinger, Julian E. Klaas

**Affiliations:** 1 Retina Vitreous Research Group, Department of Ophthalmology, University Hospital Ulm, Ulm, Germany; 2 Department of Ophthalmology, University Hospital, LMU Munich, Munich, Germany; 3 Munich Eye & Vascular Medicine, Munich, Germany

**Keywords:** Epiretinal membrane, Immunocytochemistry, Internal limiting membrane, Pseudoexfoliation syndrome, Transmission electron microscopy, Vitrectomy, Vitreomacular interface

## Abstract

**Purpose:**

To describe the morphology and histopathology of the vitreoretinal interface (VRI) in eyes with pseudoexfoliation syndrome (PEX) and epiretinal membrane (ERM) (pERM) in comparison to eyes without PEX and idiopathic ERM (iERM).

**Methods:**

Specimens of ERM and internal limiting membrane (ILM) were obtained during pars plana vitrectomy with membrane peeling from 10 symptomatic eyes with pERM and 16 eyes with iERM. Specimens were processed as flat-mounts for immunocytochemistry and prepared by ultrathin series sectioning for transmission electron microscopy (TEM). Cellular and extracellular composition were compared and correlated with clinical data.

**Results:**

The extracellular distribution of the VRI showed significant differences between pERM and iERM in the following aspects. Immunostaining revealed that all pERM specimens were positive for fibrillin-1, collagen IV and TGFβ1, which are key regulatory factors or structural components of the extracellular matrix, while these markers were rarely detected positive in the iERM group. Glial cell markers, GFAP and vimentin, were consistently positive in both groups. TEM revealed abundant vitreous collagen and evidence of vitreous remodeling in pERM eyes, including the presence of fibrous long-spacing collagen (FLSC). In contrast, iERM specimens showed sparse collagen strands with no evidence of FLSC.

**Conclusions:**

Eyes with pERM showed excessive production and subsequent accumulation of extracellular matrix material and elastic proteins such as fibrilin compared to eyes with iERM. Our data suggest that PEX may affect the VRI in a manner similar to the anterior segment, based on the detection and alteration of common structures such as collagen in the pERM.

## Abstract (highlights)

**Key messages:**

*****What is known***:**

- Recent research has associated pseudoexfoliation syndrome (PEX) with changes in the vitreoretinal interface (VRI), revealing a notably higher prevalence of epiretinal membranes (ERM) in eyes affected by PEX.

*****What is new***:**

- Our study revealed differences in the extracellular distribution of the VRI between eyes with PEX and ERM (pERM) compared to eyes without PEX and idiopathic ERM (iERM).
- All pERM samples tested positive by immunocytochemistry for fibrillin-1, collagen IV and TGFβ1, which are key regulatory factors or structural components of the extracellular matrix. Ultrastructural analysis revealed abundant vitreous collagen and signs of vitreous remodeling in pERM eyes.
- These findings indicate that PEX might affect the VRI in a way similar to its impact on the anterior segment, as shown by shared features such as the presence of collagen in pERM.

## Introduction

Pseudoexfoliation syndrome (PEX) is an age-related systemic condition that predominantly affects individuals over the age of 60, with a prevalence of up to 20% [1, 2]. Characterized by the deposition of white fibrillar material, PEX is strongly associated with an increased risk of open-angle glaucoma (OAG) and significantly harder nuclear cataracts [3, 4]. Recent studies have also linked PEX to alterations in the vitreoretinal interface (VRI) [5, 6], with significantly higher incidences of epiretinal membranes (ERM) and incomplete posterior vitreous detachment (PVD) reported in eyes with PEX [6]. Notably, the prevalence of ERMs in patients with PEX glaucoma is nearly five times higher than in those with primary OAG [5].

The development of PEX is characterized by a cascade of pathophysiological events resulting from an imbalance in extracellular matrix dynamics and oxidative stress. This condition is associated with the overproduction, impaired breakdown, and subsequent accumulation of abnormal cross-linked extracellular matrix components and elastic proteins, including elastin, fibronectin, amyloid, and fibrillin-1 [7]. Idiopathic ERMs (iERMs) are characterized by an extracellular matrix (ECM) rich in structural and adhesive components, including collagen, fibronectin, and fibrillin-1 [8]. Collagen provides mechanical stability to the membrane, while fibrillin-1 and fibronectin play key roles in cell adhesion, signaling, and ECM organization. These components contribute to the pathological remodeling of the retinal surface and are involved in the progression of iERM formation.

To date, the cellular components and extracellular composition of the VRI in patients with PEX are unknown. The aim of this study was to evaluate the cellular and extracellular distribution of the VRI in eyes with ERM associated with PEX (pERM) compared to iERMs without PEX using immunocytochemistry and transmission electron microscopy (TEM).

## Materials and methods

### Study population

From a cohort of 340 eyes that underwent pars plana vitrectomy (PPV) with peeling of the internal limiting membrane (ILM) and epiretinal membrane (ERM), ten eyes with ERM associated with PEX (pERM) were identified. For the age-matched control group, sixteen eyes with idiopathic ERM (iERM), not associated with PEX, were included. All specimens were surgically obtained at the Department of Ophthalmology, Ludwig-Maximilians-University Munich (LMU), between 2018 and 2023 and subsequently prepared for immunocytochemical analyses.

For immunocytochemical control of each of the two antibody combinations, we had two age-matched iERM controls in six cases. This was because not all samples were stained with each antibody combination due to the size of the samples. For ultrastructural analysis, we included six of ten eyes with pERM and seven of sixteens eyes with iERM as they showed homogeneous or clustered cell composition on immunocytochemistry. Specimens with single cells were not prepared for TEM.

Exclusion criteria included high myopia with more than −6.00 D, prior intraocular surgery (except for cataract surgery), history of retinal tears and retinal detachment, uveitis, vascular retinopathies or advanced cataracts that could impact the image quality of OCT data.

Recorded data comprised age, sex, presence of PEX, presence of optic atrophy, lens status before and after surgery.

Pseudoexfoliation was identified during slit-lamp biomicroscopy by the presence of a distinct white fibrillar material deposited on the surface of the crystalline lens. This material appeared either centrally or peripherally, with a darker, ring-like zone separating the two areas. Best-corrected visual acuity was documented for each patient before surgery and at the last follow-up. Optical coherence tomography (OCT) images were analyzed preoperatively, postoperatively 4 weeks after surgery, and at the final follow-up visit if additional assessments were performed. Preoperative data collection included the status of vitreous detachment, as determined by the presence of a visible vitreous membrane, the stage of the ERM according to the classification of Govetto et al. [9], the central subfield thickness (CST) and the presence of ellipsoid zone [10] defects. Postoperatively and at the final follow-up, the CST and the presence of EZ defects were assessed.

The study adhered to the ethical guidelines of the Declaration of Helsinki. Approval for surgical sample acquisition, histopathological preparation and analysis of the patients’ specimens, as well as the retrospective review of the patients’ data was obtained from the Institutional Review Board and the Ethics Committee of the Ludwig-Maximilians-University Munich (No 22–1034).

### Surgical procedure

All patients included in the study underwent a standard 23- or 25-gauge PPV. Posterior vitreous detachment (PVD) was induced by using the vitrectomy probe to apply suction around the optic nerve head. The ILM was peeled either sequentially or in one piece along with the ERM over an area of at least two-disc diameters, extending up to the vascular arcades, using end-gripping forceps. To aid in the peeling process, a 0.25 mg/mL Brilliant Blue dye solution [Brilliant Peel] (Fluoron GmbH, Neu-Ulm, Germany) was used to stain the ILM. The excised tissues were preserved in balanced salt solution (BSS) (Bausch and Lomb, Berlin, Germany) for transportation.

### Specimen preparation and immunocytochemistry

Immediately after harvesting, the specimens were immersed in a solution containing 4% paraformaldehyde (PFA) and 0.1% glutaraldehyde (GA) in 0.1 M phosphate-buffered saline (PBS, pH 7.4) for fixation. Subsequently, the specimens underwent incubation with 0.1% pepsin and were then blocked using 6% bovine serum albumin (BSA) (Sigma-Aldrich, St. Louis, US).

The following primary antibodies were utilized: anti-transforming growth factor-beta1 [anti-TGF-β1] (1:40, mouse, ab49574, ABCAM, Cambridge, UK); anti-fibrillin-1 (1:50, rabbit, SAB4500863, Sigma-Aldrich, St. Louis, US); anti-vimentin (1:40, goat, V4630, Sigma-Aldrich, St. Louis, US); anti-collagen IV [anti-coll IV] (1:50, mouse, sc-59814, Santa Cruz Biotechnology, Inc., Dallas, USA); anti-metalloproteinase 2 [anti-MMP2] (1:100, rabbit, AB19167, Merck Millipore, Billerica, Massachusetts, US) and anti-glial fibrillary acidic protein [anti-GFAP] (1:50, goat, ab49574, ABCAM, Cambridge, UK). The specimens were labeled with a combination of three primary antibodies. Following an overnight incubation with the primary antibodies at room temperature, a secondary antibody (either donkey anti-rabbit Cy2, donkey anti-mouse Cy3, or donkey anti-goat Cy5 from Dianova, Hamburg, Germany) was applied for two hours at room temperature.

Negative controls involved substituting the primary antibody with 0.1 M PBS (pH 7.4) or mouse primary antibody isotype control [IgG2a] (M5409, Sigma-Aldrich, St. Louis, US). The 4′,6-diamidino-2-phenylindole [DAPI] (AKS-38448, Dianova, Hamburg, Germany) staining was performed to visualize cell nuclei.

For flat-mount preparation, the specimens were flattened and unfolded onto glass slides to display their maximum surface area, using a stereomicroscope (MS5, Leica, Wetzlar, Germany). Phase contrast, interference microscopy, and fluorescence microscopic analyses were conducted using a modified fluorescence microscope (DM 2500, Leica, Wetzlar, Germany) at magnifications ranging from × 50 to × 400. Photographic documentation was carried out using a digital camera (ProgRes CF, Jenoptik, Jena, Germany). Two independent experienced examiners assessed the results to determine positive or negative samples.

### Transmission electron microscopy

The included specimens underwent post-fixation through a 30-min incubation with 1% OsO4 in PBS, followed by rinsing with distilled water and partial dehydration using escalating concentrations of ethanol. Next, the tissues were contrasted with 1% uranyl acetate in 70% ethanol overnight at 4 °C. This was followed by dehydration steps using 70%, 90%, and 100% ethanol (each for 20 min per change), along with two rounds of acetone (15 min each).

Ultimately, the samples were embedded in Epon and polymerized at 60 °C for 48 h. Ultrathin sections measuring 60–70 nm in thickness were examined using an EM900 transmission electron microscope at 80 keV (Zeiss, Jena, Germany). Digital micrographs were captured using a CCD-2 k camera (Troendle, Moorenweis, Germany).

### Statistical analysis

Statistical analysis was performed using IBM SPSS Statistics 29.0 Software (SPSS Inc., IBM Software Group, Chicago, IL). Descriptive statistics including mean, standard deviation (SD), median, range, minimum, maximum, and pertinent percentages were calculated. A variety of tests were used for statistical calculations, including the Wilcoxon signed-rank test and the Mann–Whitney U test. A significance level of *P* < 0.05 was used for all tests to determine statistical significance.

## Results

Demographic, functional and morphological data for the two groups are shown in Table 1.

**Table 1:** Demographic data of the epiretinal membranes associated with pseudoexfoliation (pERM) and the idiopathic epiretinal membranes (iERM)

	pERM (*n* = 10 eyes)	iERM (*n* = 16 eyes)
Age at the time of surgery [years]	*76.6* ± *4.7*	*76.7* ± *5.6*
Gender [*n*]		
male	*7 (70%)*	*10 (62.5%)*
female	*3 (30%)*	*6 (37.5%)*
Eye [*n*]		
right	*4 (40%)*	*7 (43.8%)*
left	*6 (60%)*	*9 (56.3%)*
Preoperative lens status [*n*]		
phakic	*4 (40%)*	*9 (56.3%)*
pseudophakic	*6 (60%)*	*7 (43.8%)*
Postoperative lens status [*n*]		
pseudophakic	*10 (100%)*	*16 (100%)*
Visual acuity [logMAR]		
preoperative	*0.6* ± *0.32*	*0.47* ± *0.32*
last follow up	*0.65* ± *0.43*	*0.28* ± *0.18*
Follow up [months]	*21.6* ± *15.8*	*23.6* ± *22.6*
Intraoperative status of vitreous detachment [*n*]	*n* = *9 eyes*	*n* = *16 eyes*
attached	*5 (50%)*	*1 (6.3%)*
incomplete	*1 (10%)*	*1 (6.3%)*
complete	*3 (30%)*	*14 (87.5%)*
Preoperative OCT Data	*n* = *10 eyes*	*n* = *16 eyes*
ERM Stage (Govetto et al.)		
1	*1 (10%)*	*2 (12.5%)*
2	*3 (30%)*	*3 (18.8%)*
3	*4 (40%)*	*9 (56.3%)*
4	*2 (20%)*	*2 (12.5%)*
Additional epiretinal proliferation		
yes	*0*	*0*
no	*10 (100%)*	*16 (100%)*
Visible posterior hyaloid membrane		
yes	*2 (20%)*	*1 (6.3%)*
no	*8 (80%)*	*15 (93.8%)*
Central subfield thickness [μm]	*509.6* ± *132.5*	*496.4* ± *174.5*
Ellipsoid zone defect		
yes	*2 (20%)*	*2 (12.5%)*
no	*7 (70%)*	*13 (81.3%)*
Macular edema		
MME	*4 (40%)*	*5 (31.3%)*
MME + CME	*3 (30%)*	*1 (6.3%)*
CME	*0*	*0*
no macular edema	*3 (30%)*	*10 (62.5%)*
Postoperative OCT Data (4 weeks)	*n* = *8 eyes*	*n* = *14 eyes*
Central subfield thickness [μm]	*403.6* ± *83.3*	*425.3* ± *69.9*
Ellipsoid zone defect		
yes	*2 (20%)*	*1 (6.3%)*
no	*6 (60%)*	*10 (62.5%)*
Macular edema		
MME	*3 (37.5%)*	*2 (18.8%)*
MME + CME	*0*	*1 (7.1%)*
CME	*0*	*0*
no macular edema	*5 (62.5%)*	*10 (71.4%)*
Last follow up OCT Data	*n* = *6 eyes*	*n* = *8 eyes*
Central subfield thickness [μm]	*354.8* ± *58.3*	*382.1* ± *62.3*
Ellipsoid zone defect		
yes	*2 (20%)*	*1 (6.3%)*
no	*4 (40%)*	*7 (43.8%)*
Macular edema		
MME	*1 (17%)*	*1 (12%)*
MME + CME	*0*	*0*
CME	*0*	*0*
no macular edema	*5 (83%)*	*7 (88%)*

*OCT* optical coherence tomography, *MME* microcystic macular edema, *CME* cystoid macular edema

### Demographic data

The mean age at the time of surgery was 76.6 ± 4.7 years (range, 67.1—81.7 years) in the pERM group and 76.7 ± 5.6 years (range, 66.1—85.0 years) in the iERM group. Prior to surgery, approximately half of the patients were phakic (pERM: 40%; iERM: 56.3%). All phakic patients underwent combined PPV with cataract surgery. In pERM cases, 60% presented with glaucomatous optic atrophy at baseline. Among all pERM eyes, 5 eyes (50%) were treated with anti-glaucoma eye drops. Notably, none of the pERM eyes had undergone prior glaucoma surgery. Mean follow-up time in pERM eyes was 21.6 ± 15.8 months (range, 2.7—52.3 months) and 23.6 ± 22.6 months (range, 3.0—62.8 months) in iERM controls.

### Functional and morphological results

Preoperative best-corrected visual acuity (BCVA) was 0.6 ± 0.32 logMAR in the pERM group and 0.47 ± 0.32 logMAR in the iERM group. At the last follow up, the mean BCVA was 0.65 ± 0.43 logMAR in the pERM group and 0.28 ± 0.18 logMAR in the iERM group. The difference in pre- and postoperative BCVA was not statistically significant (Wilcoxon test: pERM *p* = 1.0 and iERM *p* = 0.35). Between the two groups, BCVA was significantly better in the iERM group than in the pERM group at the last follow-up visit (Mann–Whitney U test: *p* = 0.024).

**Fig. 1:**
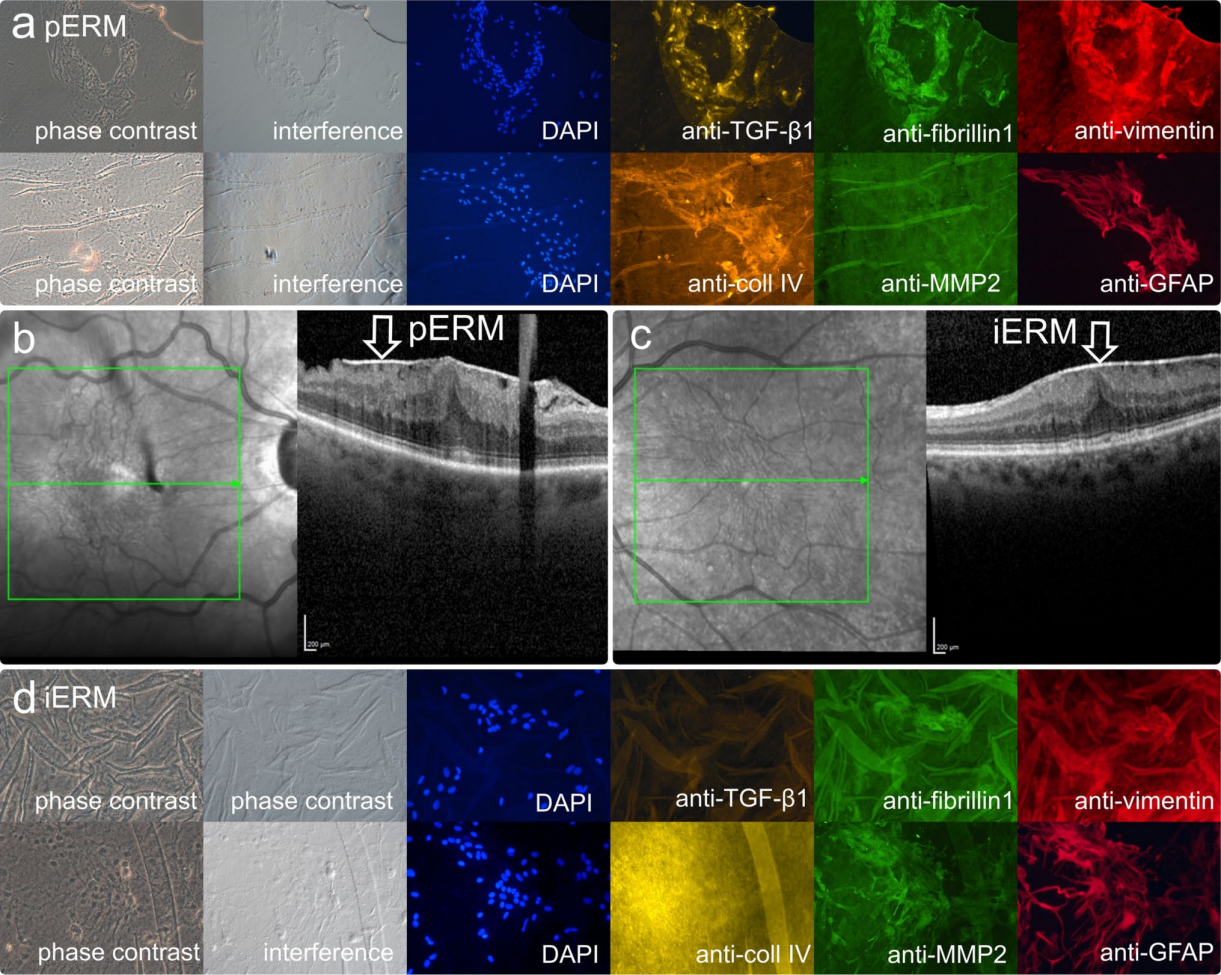
**a** Phase contrast and interference microscopy, immunocytochemical staining merged with cell nuclei staining of 4’,6’-diamidino-2-phenylindole, DAPI (blue) and fluorescence microscopy in surgically removed epiretinal membrane (ERM) associated with pseudoexfoliation (PEX) syndrome (pERM). Epiretinal cells show positive immunolabelling with anti-transforming growth factor-beta1 [anti-TGF-β1], anti-fibrillin1, anti-vimentin, anti-collagen IV [anti-coll IV] and anti-glial fibrillary acidic protein [anti-GFAP]. Negative immunoreactivity of anti-metalloproteinase 2 [anti-MMP2]. **b** Spectral-domain optical coherence tomography (SD-OCT) images of an 81-year-old male with pERM (arrow). **c** SD-OCT images of an 84-year-old female with idiopathic epiretinal membrane (iERM). **d** Phase contrast and interference microscopy, immunocytochemical staining merged with cell nuclei staining of 4’,6’-diamidino-2-phenylindole, DAPI (blue) and fluorescence microscopy in surgically removed iERM. Negative immunoreactivity of anti-TGF-β1, anti-fibrillin1, anti-vimentin, anti-coll IV and anti-MMP2. Positive immunolabelling with anti-GFAP. (Original magnification: (a, first row) × 400; (a, second row) × 200); (d) × 400)

According to the classification of Govetto et al. on OCT, the most common ERM stage observed was stage 3 (pERM: 40%; iERM: 56.3%), followed by stage 2 (pERM: 30%; iERM: 18.8%) (Fig. 1a, c). None of the eyes in either group had additional epiretinal proliferation. The majority of patients (pERM: 80%; iERM: 93.8%) had no visible posterior vitreous membrane on OCT. However, the intraoperative status of the vitreous detachment showed an adherent vitreous in more than half of the pERM eyes while a complete posterior vitreous detachment was found in 14 of 16 iERM eyes (Fisher´s exact test: *p* = 0.01).

The mean preoperative CST was 509.6 ± 132.5 µm for pERM and 496.4 ± 174.5 µm for iERM. Preoperative EZ defects were present in 20% of pERM eyes and 12.5% of iERM eyes, with a mean defect distance of 150 µm in the pERM group and 244 ± 19.8 µm in the iERM group. None of these analyzed preoperative parameters showed a statistically significant difference between the pERM and iERM group (Fisher´s exact test, Mann–Whitney U test: *p* between 0.538 and 0.820). In the pERM group, microcystic macular edema (MME) was seen in 4 of 10 eyes (40%) and combination of both MME + cystoid macular edema (CME) in 3 of 10 eyes (30%). No eyes with pERM were found to have CME. No edema was documented in 3 of 10 eyes (30%) with pERM. In the iERM group, 5 of 16 eyes (31%) showed MME and 1 of 16 eyes (6%) had combination of MME + CME. No CME was found in the iERM eyes. In 10 of 16 iERM eyes (63%) no edema was documented. There was no statistical significance between the groups (Pearson-Chi-Quadrat Test: *p* = 0.158).

At the last follow-up, the CST decreased to 354.8 ± 58.3 µm in the pERM group and 382.1 ± 62.3 µm in the iERM group. This was statistically significant in the pERM group (Friedman test: pERM *p* = 0.004 and iERM *p* = 0.055). At the final follow up, 20% in the pERM group and 6.3% in the iERM group revealed EZ defects. Postoperatively, 1 of 6 eyes (17%) in the pERM group had MME and 5 of 6 eyes (83%) presented with no edema. In the iERM group, 1 of 8 eyes (12%) had MME, and 7 of 8 eyes (88%) had no edema.

### Immunocytochemical analysis

The detailed immunocytochemical analysis of the pERM and iERM eyes is shown in Table 2.

**Table 2:** Immunocytichemical analysis of the epiretinal membranes associated with pseudoexfoliation (pERM) and the idiopathic epiretinal membranes (iERM)

Antibody	Antibody targets		pERM (*n* = 10 eyes)	iERM (*n* = 16 eyes)
TGF-beta1 [*n*]	potent regulator of cell proliferation influencing ECM interactions	positive negative	10 (100%) 0	2 (14.3%) 12 (85.7%)
Fibrillin-1 [*n*]	structural component of microfibrils in the ECM	positive negative	10 (100%) 0	8 (57.1%) 6 (42.9%)
Collagen IV [*n*] positive negative	major component of the BM in the ECM	positive negative	10 (100%) 0	4 (36.4%) 7 (63.6%)
MMP2 [*n*]	family of zinc-dependent ECM, involved in ECM remodeling and angiogenesis	positive negative	7 (70%) 3 (30%)	11 (100%) 0
Vimentin [*n*]	Müller cells, immature glial cells, astrocytes	positive negative	10 (100%) 0	10 (71.4%) 4 (28.6%)
GFAP [*n*]	glial cells, fibrous astrocytes	positive negative	9 (90%) 1 (10%)	10 (90.9%) 1 (9.1%)

TGF-beta transforming growth factor-beta, MMP Metalloproteinase, GFAP anti-glial fibrillary acidic protein, ECM extracellular matrix, BM basement membrane

In the pERM group, analysis of flat-mounted specimens showed positive immunostaining for anti-TGF-ß1, anti-fibrillin1 and anti-collagen type IV in all examined samples (Fig. 1a). The marker anti-MMP-2 was also seen positive in the majority the specimens. Anti-GFAP and anti-vimentin were positive in most samples.

In contrast, in eyes with iERM, anti-TGF-ß and anti-collagen type IV were negative in most of the samples analysed. Anti-fibrillin was positive in just over half of the samples (Fig. 1d). There was a predominance of positive immunostaining for anti-MMP-2, anti-GFAP and anti-vimentin.

In all control specimens using IgG2, no specific positive immunostaining was observed.

### Ultrastructural analysis

**Fig. 2:**
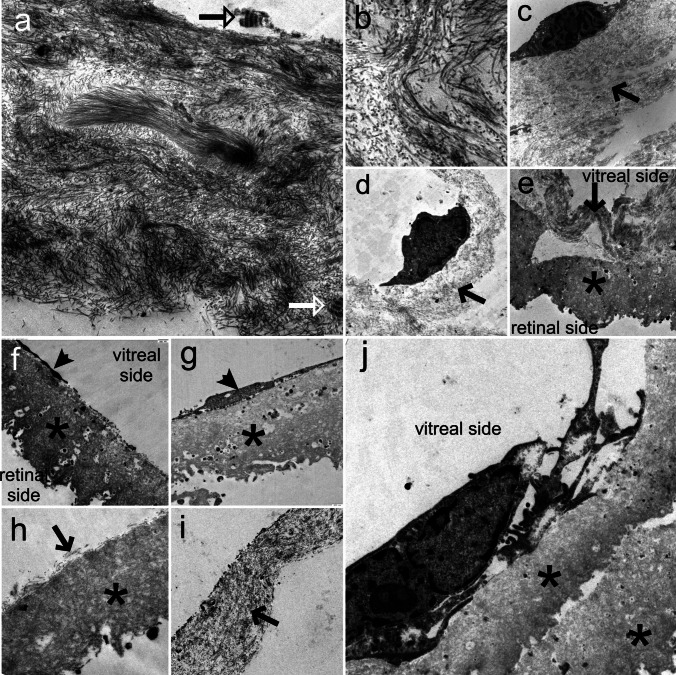
Transmission electron micrograph of removed (**a**-**e**) epiretinal membrane (ERM) associated with pseudoexfoliation (PEX) syndrome (pERM) and (**f**-**j**) idiopathic epiretinal membrane (iERM). **a** Transmission electron micrograph represents abundant vitreous cortex collagen strands and fibrous long spacing collagen (FLSC, arrows). **b** In the magnified section, the vitreous collagen fibrils are seen. **c**, **d** Myofibroblast-like cells on vitreous collagen strands (arrow). **e** The vitreous collagen strands (arrow) were identified on the internal limiting membrane (ILM, asterisk). **f**–**h** In contrast, in iERM eyes less vitreous collagen was found. **f**, **g**, **j** Cellular structures (arrowhead) were found directly on the ILM (asterisk). (**h**) ILM with single collagen fibrils on the vitreal side. **i** Strand of vitreous collagen. FLSC was not seen in iERM eyes. (Original magnification: (a) × 4400; (d) × 12 000)

Using TEM, the ILM was seen in 5 of 6 in with pERM and in 4 of 7 specimens of eyes with iERM. Characterized by its undulated retinal side and the smooth vitreal side, the ILM was clearly differentiated from attached collagen strands (Fig. 2e, f, g, h, j).

In eyes with pERM, we found amounts of vitreous collagen in the ECM in all specimens (Fig. 2a and e). It appeared with regular and irregular fibril arrangement. In addition, we also found fibrous long spacing collagen (FLSC), which is representative of remodelling processes, in 2 out of 6 eyes (Fig. 2a). Cellular structures were seen in 4 out of 6 specimens (Fig. 2c, d). Cells were identified as myofibroblasts, fibroblasts and hyalocytes at the vitreal side of the ILM. When analysing the retinal side of the ILM, we found occasional retinal fragments in all specimens with pERM. No whole retinal cells were found at the retinal ILM side.

In comparison, less vitreous collagen was seen in the iERM group (Fig. 2f and j). Notably, no vitreous collagen was seen in 3 out of 7 samples. FLSC was also not seen in any of the 7 samples. Cellular structures on the vitreal side were seen in 5 out of 7 samples, but only in 1 sample were whole cells identified as myofibroblasts and hyalocytes (Fig. 2j). In the other 4 specimens, only cell fragments were seen at the vitreal side (Fig. 2f, g). Comparable to pERM, occasional retinal fragments, but no retinal cells were found at the retinal ILM side of eyes with iERM.

## Discussion

Our study revealed differences in the extracellular distribution of the VRI between pERM and iERM. By immunocytochemistry, all pERM samples were positive for fibrillin-1, collagen IV and TGFβ1, which are important regulatory factors and structural elements of the ECM. In contrast, only half or less of the eyes with iERM were positive for these markers. Ultrastructurally, abundant vitreous collagen and signs of vitreous remodeling, including the presence of FLSC, were found in pERM eyes by TEM. Sparse collagen fibres and no evidence of FLSC were observed in iERM TEM samples.

Recently, PEX has been shown to be associated with morphological and epidemiological changes of the VRI [4, 5]. According to the available data, ERM appear to be much more frequent in eyes with PEX glaucoma than in those with primary OAG. As of now, no study is known to the authors examining ultrastructural and immunohistochemical features of ERM in eyes with PEX. The pathogenesis of PEX is strongly associated with the dysregulation of ECM components, and molecules such as fibrillin, collagen IV and TGF-β. These molecules interact in a feedback loop whereby TGF-β upregulates fibrillin and collagen IV production, leading to excessive ECM deposition and remodeling [11]. This dysregulation is central to the characteristic features of PEX, including fibrotic changes, abnormal ECM architecture, and the deposition of exfoliative material, which contributes to its known ocular complications, such as glaucoma but may also complicate PVD formation and ultimately lead to ERM formation [12].

Fibrillin is a structural glycoprotein critical for forming elastic fibers in the ECM [13]. Dysregulated fibrillin expression is associated with several diseases that share the development of fibrosis and increased cell remodeling [14]. In PEX, abnormal fibrillin production leads to the accumulation of insoluble fibrillar aggregates that are a hallmark of the syndrome [15]. This excessive deposition disrupts the structural integrity of tissues, particularly in the anterior segment, contributing to clinical manifestations such as zonular weakness and lens instability [16]. Given the findings of positive fibrillin immunoreactivity in pERM, fibrillin seems to play a major role in the formation of epiretinal cell proliferation. In iERM, it was recently shown that fibrillin-1 is widely present [17]. TGF-β1 is thought to facilitate the incorporation of fibrillin into the ECM and is mainly produced by transdifferentiated myofibroblasts [18]. The higher positivity for fibrillin-1 in pERM in our study may contribute to an increased structural rigidity and contractility compared to iERM.

Collagen IV is a primary component of basement membranes and contributes to ECM scaffolding [19]. Its overexpression in PEX is associated with the thickening and altered permeability of basement membranes. This may impair normal cell-ECM interactions and contribute to the pathological accumulation of pseudoexfoliative material.

TGF-β is a potent regulator of ECM production and remodeling playing a key role in fibrosis, potentially worsening tissue damage and dysfunction [20]. In PEX, increased TGF-β activity has been linked to excessive ECM synthesis and deposition of pseudoexfoliative material. It stimulates the production of fibrillin, collagen IV, and other ECM proteins, contributing to the accumulation of fibrillar aggregates in the anterior segment of the eye [10, 12, 18, 21]. By showing positive immunoreactivity for TGF-β1 in the pERM, our results suggest that TGF-β1 is also an important regulator of cell proliferation in the pathogenesis of ERM at VRI. Although TGF-ß1 has been associated with upregulation of MMP-2 [22], our data did not show this correlation, suggesting that MMP-2 levels may be influenced by additional regulatory mechanisms in pERM formation.

Based on immunocytochemistry and electron microscopy findings, the presence of extensive vitreous collagen strands in pERM eyes compared to iERM eyes may present challenges during vitrectomy with ERM and ILM peeling. PEX-associated pERM eyes frequently show signs of abnormal vitreous remodeling, including FLSC, which can influence the dynamics of vitreous detachment. In our study, surgeons were faced with a more adherent vitreous as confirmed by their intraoperative assessment of the vitreous status. However, in most cases of pERM, the posterior hyaloid membrane was visible on OCT as a thin, hyperreflective line just anterior to the retina. This discrepancy likely arises because surgeons assess the functional adherence of the vitreous, which may not directly correspond to OCT findings. Specifically, vitreoretinal traction or resistance can give the impression of an'attached'vitreous, even when slight separation is visible on OCT. As a result, additional surgical maneuvers are often required to achieve complete detachment, thereby increasing the complexity of the procedure. In addition, the accumulation of collagen at the VRI in pERMs likely results in greater stiffness of the membranes and retinal structures. This increased tissue rigidity may demand higher precision and force during ERM and ILM dissection and peeling, which heightens the risk of retinal trauma and surgical complications. Furthermore, stronger adhesion between the ERM, ILM and underlying retina may further complicate surgical separation and make membrane peeling more challenging.

These surgical challenges not only impact the intraoperative approach but may also have implications for postoperative recovery. Regarding the healing process, it has been suggested that the collagen-rich ECM and fibrotic environment in pERM could delay or alter the normal healing mechanisms following surgery [23]. However, in our study, we did not find significantly worse functional and structural outcomes in pERM compared to the iERM group.

Histopathological studies using immunocytochemistry and TEM provide valuable information but have several limitations. Limitations of this study include the small number of cases. These may not be representative of the wider population. However, obtaining adequate and well-preserved epiretinal tissue is challenging, especially in rare diseases such as pERM. In addition, TEM analysis was not performed on all included specimens. However, it could be argued that TEM is time consuming and requires specialised equipment and expertise, which limits accessibility and throughput. With regard to the evaluation of samples, it should be noted that immunocytochemical staining patterns may be subject to individual interpretation, introducing potential bias. Even with TEM, certain ultrastructural features, such as collagen changes, may be non-specific and observed under different conditions, making interpretation difficult.

In conclusion, our study demonstrated distinct differences in the extracellular distribution of the VRI between pERM and iERM, supported by histopathological evidence of increased production of ECM components and elastic proteins, such as fibrillin, in pERM eyes. These findings suggest that PEX may influence the VRI in a manner similar to its effects on the anterior segment, as evidenced by the presence of shared structures like collagen in pERM. Our results highlight the need for further research into clinical variations in severity, exfoliation characteristics, and postoperative outcomes in patients with pERM.
